# Measuring the quality of care in metastatic colorectal cancer: a scoping review of quality indicators

**DOI:** 10.1093/jncics/pkae073

**Published:** 2024-08-27

**Authors:** Catherine Dunn, Michael T Halpern, Daniel Sapkaroski, Peter Gibbs

**Affiliations:** Personalised Oncology Division, Walter and Eliza Hall Institute, Melbourne, Victoria, Australia; Health Sciences, The University of Melbourne, Melbourne, Victoria, Australia; Division of Cancer Control and Population Sciences, National Cancer Institute, Bethesda, MD, USA; Health Sciences, The University of Melbourne, Melbourne, Victoria, Australia; Radiation Oncology Department, Peter MacCallum Cancer Centre, Melbourne, Victoria, Australia; Personalised Oncology Division, Walter and Eliza Hall Institute, Melbourne, Victoria, Australia; Health Sciences, The University of Melbourne, Melbourne, Victoria, Australia

## Abstract

**Background:**

Quality indicators are essential for measuring and benchmarking the quality of cancer care. Although there are well-established metrics for early-stage colorectal cancer (CRC), few exist for advanced CRC. This scoping review aimed to collate and review all quality indicators for metastatic CRC.

**Methods:**

A dedicated search was performed of Web of Science, PubMed, CINAHL, and relevant gray literature to identify quality indicators for metastatic CRC, evaluating the diagnostic workup, systemic anticancer treatments, surgical approaches, radiation approaches, supportive care, and palliative or terminal care provided to patients.

**Results:**

We identified 11 articles, of which 5 were systematized reviews and 6 concerned the development, validation, or operationalization of quality indicators. Thirty-five distinct quality indicators for metastatic CRC were extracted across 6 domains of care: 1) diagnosis, staging, and treatment planning; 2) systemic anticancer treatment; 3) radiation oncology; 4) surgical approaches; 5) supportive care; and 6) palliative and end-of-life care, with a general quality indicator of overall survival. Of the 35 quality indicators extracted, 8 (23%) were unique to metastatic CRC and 27 (77%) were generic quality indicators across different tumor types but applicable to metastatic CRC.

**Conclusion:**

There are few quality indicators specifically relevant to metastatic CRC. Those that do exist are generally generic process measures used across tumor types and do not measure the nuance or complexity of current multidisciplinary treatment of patients with metastatic CRC.

Colorectal cancer (CRC) is the third-most common cancer diagnosed globally, with more than 2 million new patient in 2020 and nearly 1 million deaths from advanced disease (1). Given the rising complexity and cost of treatment, particularly for individuals with metastatic disease (2), it is important to scrutinize cancer care for consistency and quality, given the potential for adverse outcomes associated with poor-quality care.

Quality indicators are standardized, evidence-based or consensus-based measures used to assess clinical performance against agreed-upon standards or benchmarks (3). Quality indicators can be categorized using the donabedian dimensions of care (4): structural measures (the availability of necessary infrastructure, equipment, or resources), process measures (that which is actually done for or to the patient), and outcome measures (the status of a patient following an intervention, such as complication rates, quality of life or overall survival). When designing quality indicators, criteria from the US National Quality Forum dictate that for a quality indicator to be useful, it must be important (captures a significant element of care), valid (is linked to meaningful outcomes), reliable (allows credible, reproducible measurement), and actionable (informs future quality improvement endeavors). Finally and crucially, a quality indicator must be feasible (measurable using readily available, comprehensive, and contemporaneous data sources) (5).

Well-established, evidence-based clinical quality indicators exist to determine the quality of care of early-stage CRC, a disease setting characterized by discrete episodes of care that can be captured by existing administrative data (6), such as length of stay and rates of postoperative mortality (7). Existing quality indicators described by the American Society of Clinical Oncology (ASCO) (8) and the National Initiative for Cancer Care Quality (9), however, offer little insight into measuring the quality of care for patients with metastatic disease. Examples of ASCO-Quality Oncology Practice Initiative (QOPI) quality indicators include rates of recording patient consent for chemotherapy or recording patient body surface area in the medical record—crucial checklist items for patient safety but not reflective of the more complex management decisions. There is an urgent need to understand potential variability in the use of diagnostics such as imaging, pathology, and molecular testing; surgical and radiation oncology approaches; the use of systemic therapies; and integration of supportive and palliative care resources.

This scoping review establishes which quality indicators currently exist for the measurement of the quality of care given in the metastatic setting, with a focus on the feasibility and clinical utility of these quality indicators in routine clinical practice.

## Methods

### Literature search strategy

This scoping review was conducted according to Preferred Reporting Items for Systematic Reviews and Meta-Analyses for Scoping Reviews guidelines (10). A literature search was conducted between February 8 and 16, 2024, searching Web of Science, PubMed, and CINAHL for publications in English between January 1, 2014, and December 31, 2023. Search terms were constructed using Medical Subject Headings and free-text terms for CRC and quality indicators; the full search strategy can be seen in the supplementary materials (available online).

### Inclusion and exclusion criteria

We included articles examining the development, selection, review, or validation of quality indicators, including retrospective comparative cohort studies, noncomparative reviews of population-based data, data-linkage feasibility studies, expert opinion studies, and associated systematic reviews and meta-analyses.

We excluded studies of generic quality indicators for cancer care not in specific reference to CRC or if they focused solely on the screening, diagnosis, and management of early-stage CRC. Studies focused on nonclinical indicators, such as measures of cost, resource availability, or workload, were also excluded.

### Data extraction and data synthesis

Articles were searched for unique quality indicators in metastatic CRC across the spectrum of disease, from diagnosis of metastatic disease to death, recording a short description, relevant discipline (radiology, pathology, surgical, radiation oncology, medical oncology, supportive or palliative care), domain of care (diagnosis, staging and treatment planning, medical management, surgical management, supportive care or terminal care), the numerator, denominator, data sources, rationale, risk adjustment, potential barriers to operationalization, and the donabedian classification (structural measure, process measure, or outcome measure). Similar quality indicators were grouped and given a single descriptor and the most representative numerator and/or denominator.

Articles underwent citation and reference searching, and we reviewed the relevant gray literature from national bodies, including ASCO and ASCO-QOPI, the National Comprehensive Cancer Network (NCCN), and the National Initiative of Cancer Care Quality. Results were downloaded and imported into Covidence software (Covidence, Melbourne, Victoria, Australia) to remove duplicates, screened first by title and abstract, and retrieved for full text review. Papers were screened by first author C.D., with queries subject to a discussion with co-author D.S. until consensus was reached. In cases where consensus could not be reached, a third reviewer (P.G.) was consulted to provide an independent and final assessment.

Each paper describing the development or implementation of quality indicators was reviewed against the Standards for QUality Improvement Reporting Excellence 2.0 appraisal tool (11) for methodological rigor, including review of sample size, data completeness, bias, reliability and replicability. The systematic reviews of quality indicators were assessed against the Assessing the Methodological Quality of Systematic Reviews 2 tool to assess their search strategy and data-extraction techniques (12). These studies were reviewed for the number of quality indicators included in each paper; the proportion dedicated to CRC compared with other solid organ tumor types; those describing quality in metastatic vs early-stage disease; and accompanying data regarding quality indicator measurability, clinical utility, and barriers to use.

## Results

### Included studies

**Figure 1.**
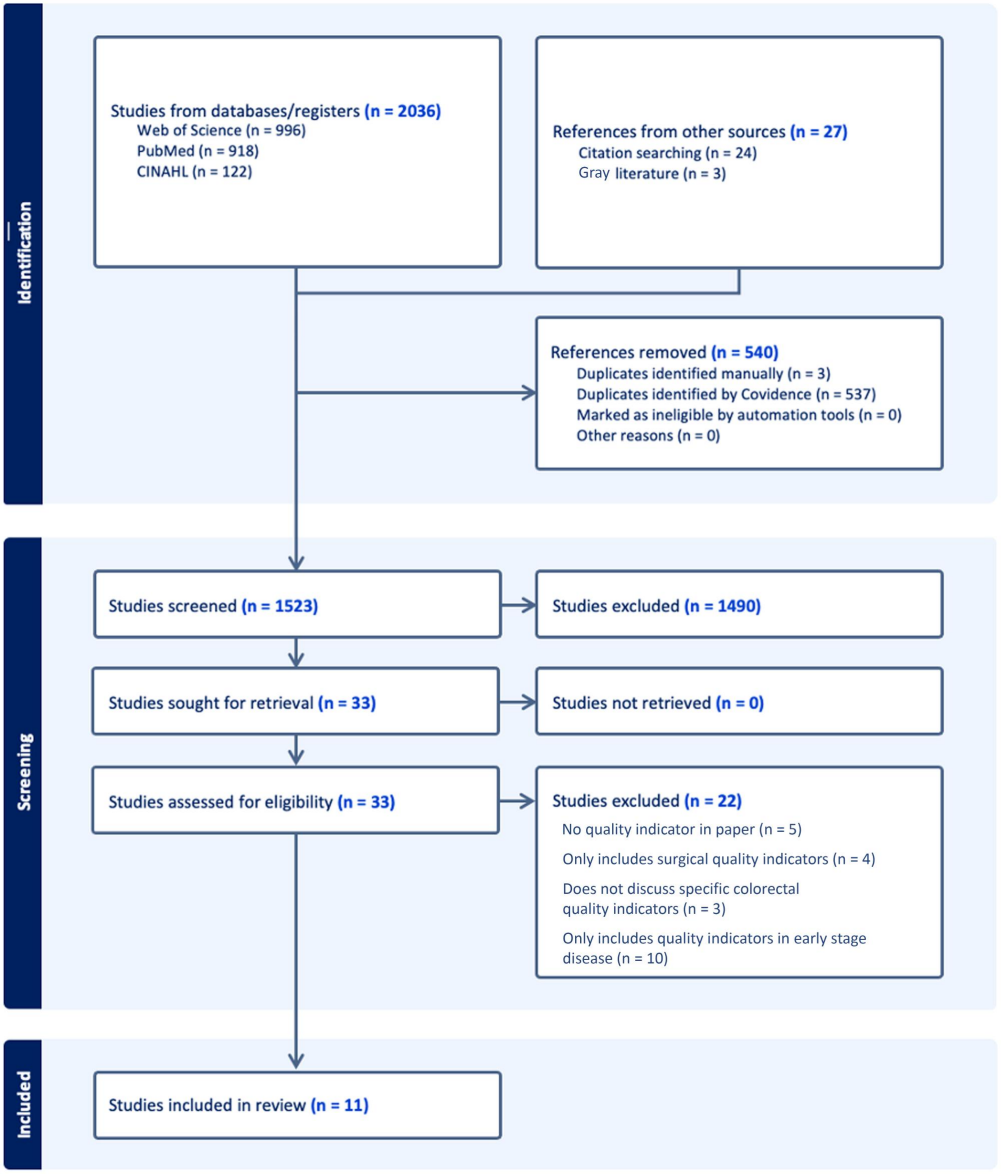
Preferred Reporting Items for Systematic Reviews and Meta-Analyses diagram of search strategy, screening, and extraction

**Figure 2.**
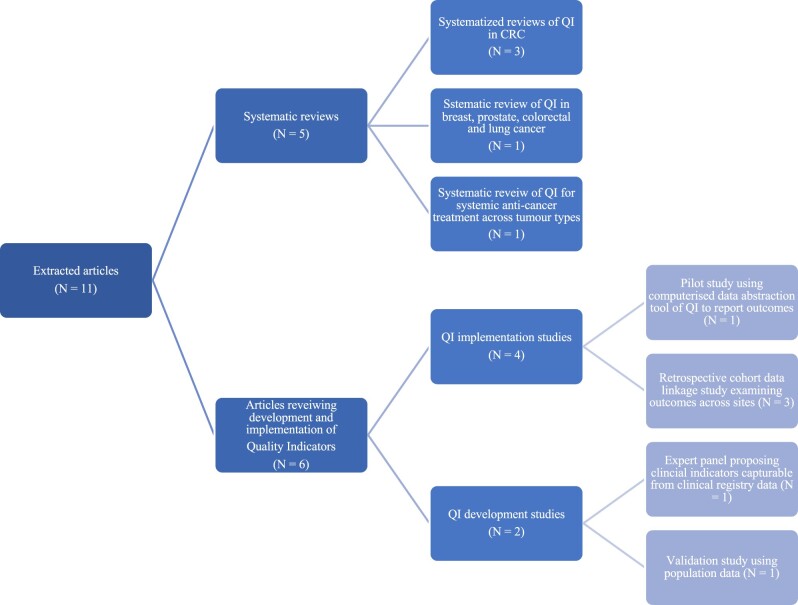
Characteristics of extracted articles. CRC = colorectal cancer.

The literature search yielded 2036 articles, with a further 27 identified from citation and reference searching. After removing duplicates, screening, and full text review, 11 articles were included in the final analysis (Figure 1). Of those 11 articles, 5 were systematized reviews: 3 reviewed quality indicators specific to CRC; 1 reviewed quality indicators in lung cancer, breast cancer, prostate cancer, and CRC; and 1 evaluated quality indicators of systemic anticancer treatment across all solid organ malignancies. The remaining 6 articles concerned the development, validation, or operationalization of quality indicators (Figure 2).

All articles were from high-income countries, including from the United States (n = 2 [18%]), Canada (n = 2 [18%]), the United Kingdom (n = 2 [18%]), Australia (n = 3 [27%]), the Netherlands (n = 1 [9%]), and an international collaboration from the International Consortium for Health Outcomes Measurement (n = 1 [9%]).

### Data sources

Three major approaches were seen in the 6 studies of quality indicator development and implementation.

#### Data linkage studies

Of the 6 studies detailing the development and implementation of quality indicators, 3 detailed linkage of robust, preexisting, population-based datasets, including linking of cancer registry data, admission data, *International Statistical Classification of Diseases, Tenth Revision* codes, and mortality data (13-15). These studies compared quality indicators between institutions to identify outliers. A strength of these studies was their large numbers, allowing case mix adjustment and robust analysis of associations between the quality indicator and both patient-level factors (eg, age, gender, insurance status, and socioeconomic status) and hospital-level factors (eg, public and private, rurality, caseload). An important limitation to each of these studies, however, was the uncertainty about the completeness and accuracy of data, which may differ between institutions.

#### Manual chart abstraction

Two studies outlined approaches dependent on manual chart abstraction for complex clinical and pathological data, both using purpose-built computerized data-abstraction tools administered by trained abstractors (16,17) outside of existing administrative datasets or workflows.

#### Dedicated cancer registries

Finally, 1 paper outlined the development of consensus-based quality indicators specific to the care of patients with metastatic CRC using data routinely captured in a comprehensive clinical registry (18). This study was limited by a smaller sample size than population-based efforts, and the authors described limitations in data completeness for some metrics (including timing of administration of chemotherapy after diagnosis and before death and referral to palliative care). Therefore, some proposed quality indicators were not evaluable and would require data linkage with larger administration datasets.

### Studies examining quality indicator development or implementation

Of the 6 studies examining the development or implementation of quality indicators (Table 1), 5 (83%) focused purely on CRC and 1 described a quality indicator of chemotherapy toxicity across all cancer types (14). Of those studies just examining CRC, 3 included quality indicators across all phases of the disease, 1 study detailed quality indicators using data captured in a metastatic CRC registry (18), and 1 study described quality indicators specific to the terminal phase (15).

**Table 1.** Characteristics of extracted studies: articles about quality indicator development or validation^a^

Article	Country	Tumor streams	Patients, No.	Sites, No.	Quality indicators, No.	Metastatic CRC quality indicators, No.	Study design	Strengths and limitations
Jackson, 2013 (16)	United States	Colorectal	1373	28	31	6	Descriptive study of implementation of computerized data-abstraction tool to measure outcomes across institutions	Resource-intensive manual data abstraction from clinical record Limited data completeness
Hu, 2014 (15)	Canada	Colorectal	2074	17	5	5	Retrospective data linkage cohort study to assess variability in end-of-life care between regional hospitals	Comprehensive, high-quality datasets Appropriate case adjustment and multivariate analysis
Jorgensen, 2014 (13)	Australia	Colorectal	6890	17	11	1	Retrospective data linkage cohort study from cancer registry, administration data, and death registry to assess variability in quality indicators across hospitals	Comprehensive, high-quality datasets Appropriate case adjustment and multivariate analysis
Siegal, 2014 (17)	United States	Colorectal	1000	10	35	5	Descriptive study of computerized data-abstraction tool to prospectively measure outcomes across institutions at 2 time points to assess change in quality indicator adherence	Resource-intensive manual data abstraction from clinical record High-quality data and stringent quality control
Turner; 2015 (18)	Australia	Colorectal	1276	10	13	13	Retrospective registry data analysis to compare hospital quality indicator adherence	Comprehensive dataset covering clinical and pathological variables relevant to CRC quality indicators Comparatively smaller numbers to compare between sites Lacking hospital administrative data
Boyle, 2023 (14)	United Kingdom	All cancers	7683	106	1	1	Retrospective data linkage study to assess variability in the composite measure of chemotherapy toxicity between UK National Health Service hospitals	Comprehensive, high-quality datasets Appropriate case adjustment and multivariate analysis

a CRC = colorectal cancer.

The studies were heterogenous in their contexts and quality indicator development strategy. The size of the studies varied from 1000 to 7683 patients (median = 1723 [IQR = 1207-7088]), and the number of institutions involved varied from 10 to 106 (median = 22 [IQR = 10-105]). All were retrospective cohort studies or based on expert consensus (Oxford level 4 and level 5 evidence) (19).

The studies were diverse regarding both geography and funding sources, which made extrapolation across clinical settings challenging. One study exclusively examined outcomes for US veterans (16), and another studied outcomes for insured patients across institutions in Florida (17). In contrast, papers from the United Kingdom and Canada (14,15) examined settings with universal health care, and the 2 Australian papers incorporated public and private health-care settings (13,18).

One study used an expert panel to identify potential “evidence-based” or “best practice–based” quality indicators that could be captured from an existing clinical registry (18). This study used existing clinical CRC cancer data-collection efforts, incorporating clinicopathological characteristics of all patient throughout the spectrum of the disease.

Another study piloted the use of a computerized data-abstraction system to evaluate performance against 31 quality indicators based on the NCCN guidelines (16) across 28 institutions in the United States, detailing important challenges in the manual abstraction of clinical data to populate complex or nuanced quality indicator items and the difficulties of reliably capturing events occurring in external institutions. Modifying existing quality indicators for lung cancer, breast cancer, prostate cancer, and CRC from ASCO-QOPI (8,9), the Florida Initiative for Quality Cancer Care reported the process of manual chart abstraction for 1000 patients across 10 sites, assessing interval change in quality indicator adherence across 2 reporting periods (17). These data included all eligible CRC patient across sites within the 2-year period, with comprehensive data-abstraction processes, quality control, and case mix adjustment.

Finally, 3 large retrospective cohort studies (13-15) reported implementation of quality indicators using comprehensive population-based datasets, using complex data linkage between clinical, administrative, and registry data to enable analysis of patient-level and hospital-level factors associated with variable quality indicator adherence or outcomes.

### Systematic reviews of quality indicators

Of the 5 prior papers detailing comprehensive literature reviews or performing a complete systematic review (Table 2), the 2017 meta-analysis from Keikes et al. (20) defined 389 discrete quality indicators from 41 studies across all stages of CRC. This meta-analysis categorized quality indicators by donabedian category and discipline and analyzed their scientific credibility (consensus-based, evidence-based, or validated in well-conducted cohort studies) but notably did not identify any established evidenced-based or validated indicators for metastatic CRC.

**Table 2.** Characteristics of extracted studies: articles reviewing quality indicators—systematic reviews and modified Delphi studies^a^

Article	Country	Tumor streams	Papers, No.	Quality indicators, No.	Metastatic CRC quality indicators, No.	Study design and aims	Conclusions
Khare, 2016 (23)	Canada	Colorectal, breast, prostate, lung	N/A	24 (colorectal) 20 (lung) 19 (prostate) 19 (breast)	5	Literature review of quality indicators in lung, breast, colorectal, and prostate cancers 3-phase modified Delphi process involving a multidisciplinary expert panel and consensus indicator set	Did not detail the search strategy of literature review List prioritized indicators that reflected high-quality care rather than feasibility List of indicators requires data not currently reliably collected
Keikes, 2017 (20)	Netherlands	Colorectal	41	389	N/A	Systematic review of existing quality indicators in CRC across the disease spectrum Characterization of quality indicators through donabedian domain, discipline and evidence base	Majority of quality indicators are surgical process measures Majority of quality indicators have a limited evidence based and have not been validated against outcomes No reported evidence-based indicators in metastatic CRC
Zerillo, 2017 (24)	International Consortium for Health Outcome Measurement	Colorectal	310	31	7	Systematic review of specifically patient-centered quality indicators Two-step modified Delphi review to select QI and case mix variables for inclusion.	Value-based quality indicator set developed, including multiple patient-reported outcome measures Requires data collection beyond the capabilities of most institutions but will be facilitated by improvements in electronic health records and redesign of clinical workflows
Donnelly, 2023 (22)	Australia	Colorectal	118	28	5	Systematic review of existing quality indicators in CRC across the disease spectrum Two-step modified Delphi review to select quality indicators for indicator set relevant to delivery of multidisciplinary cancer care in Australia	Prioritization of evidenced-based and multidisciplinary indicators No focus on feasibility
Leung, 2023 (21)	United Kingdom	All streams	15	63	7	Systematic review of existing quality indicators to examine quality of systemic anticancer treatment administration	Majority of quality indicators for systemic anticancer treatment were process measures for guideline-concordant care Very few quality indicators to capture tumor-specific elements of care

a CRC = colorectal cancer; N/A = not applicable; QI = Quality Indicator.

The second systematic review analyzed existing quality indicators, evaluating systemic anticancer treatment across all cancer types (21). This review identified 63 unique quality indicators, mostly process-based indicators focused on the appropriateness and safety of prescribed therapies based on NCCN and ASCO-QOPI guidelines (8,9). Notably, this study focused on the care of the 4 most common tumor types: breast cancer, lung cancer, prostate cancer, and CRC. Of the 63 quality indicators in this meta-analysis, 7 were generic indicators for metastatic disease, but there were no quality indicators specifically for metastatic CRC.

Finally, 3 (60%) of the extracted studies detailed a systematic review of the literature to identify potential quality indicators before an expert panel or focus group refined them, using a modified Delphi approach to reach a consensus set for use in their dedicated health-care setting (22-24). Each of these approaches constructed a comprehensive list of quality indicators, but none incorporated feasibility as a prerequisite for selection. One such study (23) determined that the data required for its proposed indicator set was not currently collected by existing administrative datasets and would be variably documented in medical records, thus limiting manual abstraction. Moreover, the International Consortium for Health Outcomes Measurement explicitly acknowledges that its indicator set requires complex structured clinicopathological data that would “stretch the capabilities of most institutions” (24).

### Extracted quality indicators in metastatic CRC

**Figure 3.**
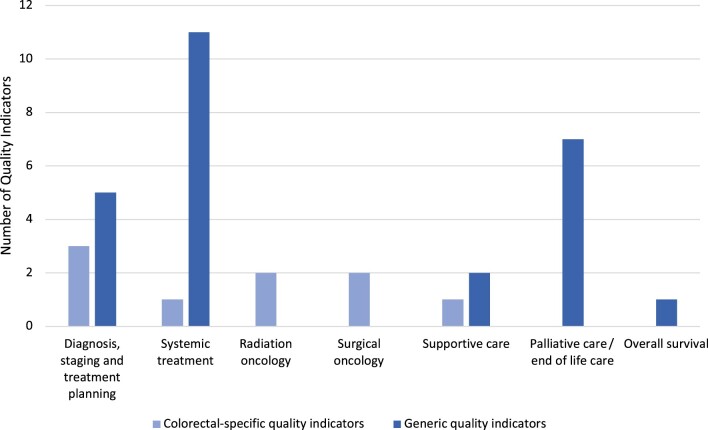
Quality indicators across domains of care.

Thirty-five distinct quality indicators for the metastatic setting were extracted from the 11 studies across 6 domains of care: 1) diagnosis, staging, and treatment planning; 2) medical oncology and systemic anticancer treatment; 3) radiation oncology; 4) surgical approaches; 5) supportive care; and 6) palliative and end-of-life care, with a general quality indicator of overall survival. Of the 35 quality indicators extracted, 8 (23%) were unique to metastatic CRC and 27 (77%) were generic quality indicators across different tumor types but applicable to metastatic CRC (Figure 3). The details of the extracted quality indicators, including description, rationale, numerator, denominator, and potential data sources, are outlined in Table 3.

**Table 3.** Characteristics of extracted quality indicators^a^

Characteristic	Reference	Rationale	Donabedian category	Generic or specific to CRC	Numerator	Denominator	Data source	Issues or limitations
**Diagnosis and treatment planning quality indicators**								
Recurrence detected on surveillance	(8,18,17)	Early relapse detection may permit curative resection	Outcome measure	Specific to CRC	No. of patients with metachronous metastatic CRC detected on surveillance schedule (carcinoembryonic antigen, colonoscopy, imaging)	No. of patients with metachronous metastatic CRC	Clinical cancer registry	—
Pathology report in file	(14,23)	Patient safety and diagnostic ascertainment	Process measure	Generic	No. of patients with pathology report in medical record	Total No. of patients with metastatic CRC	Abstraction from medical records	—
Documented staging	(8,17) ASCO-QOPI	Patient safety and diagnostic ascertainment	Process measure	Generic	No. of patients with pathology report in medical record	Total No. of patients with metastatic CRC	Abstraction from medical records	—
Mismatch repair testing	(18)	Guides treatment decisions and screens for hereditary cancer syndromes	Process measure	Specific to CRC	No. of patients with mismatch repair genes tested	Total No. of patients with metastatic CRC	Abstraction from medical records Clinical cancer registry	—
*KRAS* variation testing	(18)	Guides treatment decisions	Process measure	Specific to CRC	No. of patients with *KRAS* tested	Total No. of patients with metastatic CRC	Abstraction from medical records Clinical cancer registry	—
Case discussion in multidisciplinary team	(13)	Multidisciplinary team discussions improve diagnostic and staging practice	Process measure	Generic	No. of patients discussed in multidisciplinary team	Total No. of patients with metastatic CRC	Abstraction from medical records Hospital administrative or coding data Clinical cancer registry	Does not determine whether discussion is robust or implemented
Time between diagnosis and treatment, by modality	(18,16,23)	Measure of health-care efficiency and patient experience	Outcome measure	Generic	—	—	Abstraction from medical records Hospital administrative or coding data Clinical cancer registry	Often requires data linkage Challenge to determine accurate date
Time between pathological diagnosis and informing patient	(16) NCCN	Measure of health-care efficiency and patient experience	Outcome measure	Generic	—	—	Abstraction from medical records	Often requires data linkage Challenge to determine accurate date
**Systemic treatment quality indicators**								
Referral to a medical oncologist	(9,21) National Initiative for Cancer Care Quality	Guideline concordant care	Process measure	Generic	All patients with a diagnosis of metastatic CRC referred to a medical oncologist	All patients with a diagnosis of metastatic CRC	Abstraction from medical records Coding data	—
Recording of body surface area	(9) National Initiative for Cancer Care Quality/ASCO-QOPI	Patient safety	Process measure	Generic	No. of patients who received a chemotherapy with body surface area recorded	No. of patients who received chemotherapy	Abstraction from medical records	—
Recording of consent for chemotherapy	(9,17,21,23) National Initiative for Cancer Care Quality/ASCO-QOPI	Patient safety	Process measure	Generic	No. of patients who received chemotherapy with consent recorded	No. of patients who received chemotherapy	Abstraction from medical records	—
Chemotherapy flow sheet, notes, and blood results	(17,23,9,21) National Initiative for Cancer Care Quality/ASCO-QOPI	Patient safety	Process measure	Generic	No. of patients who received a chemotherapy flow sheet	No. of patients who received chemotherapy	Abstraction from medical records	—
Recommendation for chemotherapy where guidelines suggest it	(16,17) NCCN	Guideline-concordant care	Process measure	Generic	No. of patients who had chemotherapy discussed where appropriate	No. of patients appropriate for chemotherapy	Abstraction from medical records Clinical cancer registry	Complex clinical detail required
Evidence-based regimen of chemotherapy administered	(18,21,22) National Initiative for Cancer Care Quality	Guideline-concordant care	Process measure	Generic	No. of patients who had appropriate chemotherapy consistent with dose and cycle guidelines	No. of patients receiving chemotherapy	Abstraction from medical records Chemotherapy prescribing software Clinical cancer registry	Challenges in determining suitability in regimen or dosing
Chemotherapy in older patients	(18)	Older patients are at risk of undertreatment	Process measure	Specific to CRC	No. of patients >75 y of age who receive chemotherapy	No. of patients >75 y of age	Abstraction from medical record Chemotherapy prescribing software Clinical cancer registry	—
Use of antiemetics	(18,21,22) National Initiative for Cancer Care Quality	Guideline-concordant care	Process measure	Generic	No. of patients on highly emetogenic chemotherapy receiving a serotonin antagonist and a steroid	No. of patients on high emetogenic chemotherapy	Abstraction from medical records Chemotherapy prescribing software	Complex clinical detail required
Use of granulocyte colony stimulating factor	(18,21,22) National Initiative for Cancer Care Quality	Guideline concordant care	Process measure	Generic	Patients with >40% chance of neutropenic fever or prior episode of neutropenia	All patients treated with granulocyte colony stimulating growth factor	Abstraction from medical records Chemotherapy prescribing software	Complex clinical detail required
Treatment toxicity	(14,21,23,24)	Health-care utilization and patient experience	Outcome measure	Generic	Complication leading to hospitalization, discontinuation of treatment, limitations to functioning, or death	All patients	Abstraction from medical records Coding data Purpose built patient-reported outcomes measurement tool	—
Rates of chemotherapy-associated peripheral neuropathy	(24)	—	Outcome measure	Generic	Tracked by serial European Organisation for Research and Treatment of Cancer Quality of Life Questionnaire-liver metastases	—	Abstraction from medical records Chemotherapy prescribing software Purpose built patient-reported outcomes measurement tool	Time-consuming data collection
Rates of clinical trial participation	(18,23)	—	Outcome measure	Generic	Patients in a clinical trial	All patients	Abstraction from medical records Clinical cancer registry	—
**Radiation oncology quality indicators**								
Quality of life following radiation therapy	(24)	—	Outcome measure	Specific to CRC	Tracked by serial European Organisation for Research and Treatment of Cancer Quality of Life Questionnaire 29 and Memorial Sloan Kettering Cancer Center Bowel Function Diary	—	Abstraction from medical records Purpose-built patient-reported outcomes measurement tool	Time-consuming data collection
Radiation oncology consult for all rectal cancers	(17,22) NCCN	Guideline-concordant care	Process measure	Specific to rectal cancer	No. of patients referred for consultation with radiation oncology	Total No. of patients with CRC	Abstraction from medical records Coding data	Difficult to determine if not recorded or declined by patient
**Surgical management of metastatic disease quality indicators**								
Resection of isolated liver metastases	(18,22)	Potentially curative approach with proven survival benefit	Process measure	Specific to CRC	No. of patients with oligometastatic liver cancer who underwent resection	No. of patients with isolated liver metastases	Abstraction from medical records Clinical cancer registry Coding data	Needs detailed clinical data to accurately determine denominator
Resection or ablative therapy to isolated lung metastases	(16,18,22) NCCN	Established treatment paradigm	Process measure	Specific to CRC	No. of patients with oligometastatic lung cancer who underwent resection or ablation	No. of patients with isolated lung metastases	Abstraction from medical records Clinical cancer registry Coding data	Needs detailed clinical data to accurately determine denominator
**Supportive care quality indicators**								
Discharge letter details treatment summary, follow-up, and monitoring	(22)	Communication and care planning	Process measure	Generic	No. of patients with a discharge letter stating details of treatment, follow-up, and monitoring provided to the patient on day of discharge and referring clinician within 4 wk	All patients	Abstraction from medical records	—
Referral for genetics if positive family history	(23)	—	Process measure	Specific to CRC	No. of patients with family history of CRC referred to genetics	No. of patients with family history of CRC	Abstraction from medical records	Complex data extraction
Quality of life	(24)	—	Outcome measure	Generic	Outcomes on European Organisation for Research and Treatment of Cancer Quality of Life Questionnaire 30 or 29	—	Abstraction from medical records Purpose-built patient-reported outcomes measurement tool	Time-consuming data collection
**Palliative or end-of-life care quality indicators**								
Referral to palliative care	(18,24)	Early involvement of palliative care is associated with improved survival and quality of life	Process measure	Generic	—	—	Abstraction from medical records Administration/claims data	Often requires data linkage
Chemotherapy in final 14, 30, or 90 d of life (%)	(15,18,22)	Overly aggressive care and possibly poor palliative care	Outcome measure	Generic	Patients who receive chemotherapy 14 or 30 d before death	Decedents	Chemotherapy prescribing software Abstraction from medical records Clinical cancer registry Coding data	Often requires data linkage
Radiation therapy in final 30 or 90 d of life (%)	(15,22)	Overly aggressive care and possibly poor palliative care	Outcome measure	Generic	Patients who receive radiation therapy 30 or 90 d before death	Decedents	Radiation therapy software Abstraction from medical records Clinical cancer registry Coding data	Often requires data linkage
>1 Emergency department visit in last month of life (%)	(15)	Implied lack of palliative care services or aggressive end-of-life care	Outcome measure	Generic	Patients who present to an emergency department in the final month of life	Decedents	Abstraction from medical records Coding or administrative data	No clear link between aggressive measures of care and quality of care
>1 Hospital admission in the last month of life	(15,24)	Implied lack of palliative care services or aggressive end-of-life care	Outcome measure	Generic	Patients admitted to hospital in final month of life	Decedents	Abstraction from medical records Coding or administrative data	No clear link between aggressive measures of care and quality of care; unable to discern nature of hospital admission or quality of care received
>1 Intensive care unit admission in the last month of life	(15)	Implied aggressive end-of-life care	Outcome measure	Generic	Patients admitted to intensive care unit in the final month of life	Decedents	Abstraction from medical records Coding or administrative data	—
Death in an acute-care bed	(15,24)	Implied lack of palliative care services	Outcome measure	Generic	Patients dying in acute-care bed	Decedents	Abstraction from medical records Coding or administrative data	Unable to discern nature of hospital admission, patient preference, or quality of care received
**Overall outcome measure**								
Median overall survival or 5-y overall survival	(18,23)	—	Outcome measure	Generic	All patients	All patients	Clinical cancer registry data Administrative or death data	—

a ASCO-QOPI = American Society of Clinical Oncology Quality Oncology Practice Initiative; CRC = colorectal cancer; NCCN = National Comprehensive Cancer Network.

Systemic anticancer treatment made up the largest group of quality indicators (n = 12 [34%]), none of which were specific to CRC. The majority (10/12 [83%]) were process measures, such as recording body surface area, consent, blood results, and administration of supportive medications such as antiemetics and granulocyte colony stimulating factor. These quality indicators reflect accepted minimum standards for safe chemotherapy administration in ASCO and NCCN guidelines and are established quality indicators in the ASCO-QOPI (8). None addressed specific chemotherapeutic approaches, targeted therapies, or immune therapies used in metastatic CRC.

The next-largest group pertained to palliative and end-of-life care, all generic outcome measures of the use of high-acuity health-care resources toward the end of life (n = 9), such as chemotherapy, radiation therapy, emergency department visits, intensive care unit admissions, and use of acute-care beds (15).

Quality indicators pertaining to diagnosis, staging, and treatment planning comprised both process and outcome measures. These measures included generic process measures across solid organ malignancies (documentation of pathology, staging, and multidisciplinary team discussion) as well as process measures unique to CRC (2 quality indicators measuring rates of molecular testing for mismatch repair and *RAS* variations). Notably, these quality indicators do not reflect the breadth of molecular diagnostics now used routinely. Two quality indicators described generic time-based outcome measures designed to capture efficiency of care and the patient experience (time between diagnosis and treatment and time between histological diagnosis and informing the patient).

There were smaller numbers of quality indicators for radiation oncology (n = 2) and surgical oncology (n = 2), all specific to metastatic CRC. This finding likely pertains to the exclusion of quality indicators intended to assess screening and the definitive treatment of early-stage disease.

## Discussion

Health services research is conducted across diverse settings, with variable contexts, funding sources, and stakeholders, making comparison and extrapolation challenging (25). Nevertheless, measuring the quality of clinical cancer care is of the utmost importance, and the scope of the review and the inclusion criteria were purposefully broad to capture all possible quality indicators across a range of settings.

The majority of quality indicators found were process measures focused on discrete markers of safety and quality (eg, was compliance with a specific recommendation—such as chemotherapy consent or documentation of body surface area—seen in the clinical notes?). Although endorsed by national bodies such as ASCO and NCCN, the relationship between such process measures and clinical outcomes is uncertain, and it is challenging to determine whether such indicators reflect improved performance or simply improved documentation (4). In contrast, outcome measures capture health states, conditions, or patient-reported measures, but it is difficult to determine what variability in these outcome measures may represent. For example, outcome-based quality indicators focused on health-care utilization at the end of life, such as time spent in hospital, may reflect inappropriately aggressive anticancer care in the terminal phases of the disease (26) or the availability of inpatient palliative care systems or patient preferences for their use. The interpretation of such quality indicators is highly dependent on the clinical context.

Next, there is the difficulty of examining the quality of care in sufficient detail and granularity, with limitations on available data and frequent lags in data reporting and analysis. For example, the quality indicators specific to CRC (rates of standard molecular testing, identification of genetic syndromes, ablative approaches to oligometastatic disease) currently encompass only a fraction of the nuance and complexity of the modern multidisciplinary approach (27). This finding reflects the delays in quality indicator development, integration into data management systems, and eventual quality reporting. Moreover, the routine use of these quality indicators is limited because they require complex data extraction by dedicated personnel or comprehensive cancer registries to capture such clinicopathological detail. This challenge is only expanding as cancer care is increasingly personalized and relevant to ever-smaller patient subsets, which limits robust comparison. For example, to clarify whether patients are receiving appropriate, guideline-directed therapy, the population studied must be refined by primary site, molecular subtype, line of therapy, performance status, and patient preference for or against therapy (28). Similarly, the quality indicators capturing patient-reported outcomes, such as health-related quality of life, although valuable and desired by consumers, require questionnaires not usually incorporated into busy clinical settings, as outlined in the paper from the CRC working group of the International Consortium for Health Outcomes Measurement (24).

Finally, there is the challenge of capturing sufficient data to detect notable differences in outcomes between populations or practices. Outcome measures can be reliably captured only by large population-based datasets or large prospective studies, routinely and systematically collected and amenable to case mix adjustment—such as those of the UK National Health Service; the Surveillance, Epidemiology and End Results Program or National Cancer Database in the United States; or similarly large population-based cancer registries seen in Canada or Europe (15,29). Such data may lack the granularity to identify the cause of any disparity between sites, however, or to enable dedicated improvement efforts. Given the multiple health-care settings and stakeholders involved, selection of appropriate quality indicators will necessarily differ by scenario. Our search excluded studies not published in English and that captured only health services research in high-income countries, which is a limitation of our study and reduces generalizability.

Although this scoping review has identified the heterogeneity of existing quality indicator research in CRC, it has also highlighted the fundamental lack of quality indicators to capture and evaluate the nuance of the metastatic phase of the disease in necessary detail. For example, only 2 CRC-specific quality indicators examined the breadth of information required to adequately characterize the molecular status of a patient needed to guide treatment selection—likely a substantial deficit given the expanding treatment armamentarium, including chemotherapy, immunotherapy, and targeted therapies (30). It is essential that optimal treatment be given to well-defined patient subsets to ensure high-quality care, with minimization of cost and toxicity (31). Emerging CRC quality indicators must therefore focus on patient selection, sequencing of treatments, and integration of modern diagnostic aids—imaging and omics included—to truly reflect best practice and value-based care. Finally, in an era of increasing patient input and collaboration, the design and implementation of quality indicators should involve effective consumer consultation.

In summary, this review identified an abundance of generic process measures for CRC, clustered around the diagnostic and palliative phases of care. Renewed effort is essential to develop appropriate and meaningful quality indicators focused on the nuance and detail of clinical practice in this complex and costly phase of the disease. Although a major challenge of health services research has been capturing the requisite data to reliably measure health-care outcomes and empower future quality endeavors, there is every reason to be optimistic that big data will be transformative and that future work will focus on barriers and enablers to routine data collection. As sophisticated informatics is increasingly integrated into routine service delivery, including much-needed elements such as patient-reported outcomes, we can develop standardized quality indicators that truly reflect the quality of cancer care, benchmark across sites, and identify meaningful opportunities for improvement.

## Supplementary Material

pkae073_Supplementary_Data

## Data Availability

No new data were generated for this scoping review, but the full search strategy is available as an appendix.
