# Supplementary material for: Measuring the quality of care in metastatic colorectal cancer: a scoping review of quality indicators
Source: JNCI Cancer Spectr. 2024 Aug 27;8(5):pkae073. doi: 10.1093/jncics/pkae073 (PMC11487155; doi:10.1093/jncics/pkae073)
Supplement: pkae073_Supplementary_Data [file pkae073_supplementary_data.docx]

Appendix 1 - Search Strategy

**What are the existing quality indicators (QIs) to measure the quality of care in metastatic colorectal cancer (mCRC) from diagnosis to death?**

Search terms – used for Web of Science and adapted for PubMed and CINAHL.

| Topic 1 – metastatic colorectal cancer | MeSH terms – colorectal neoplasms (MeSH)  String terms - “colorectal neoplas*” OR “colon cancer” OR “rectal cancer” OR "colon carcinoma" OR "rectal carcinoma" OR "colorectal carcinoma" OR “colorectal tumo?r*” OR “rectal tumo?r” OR “sigmoid cancer” OR “sigmoid tumo?r” OR “sigmoid carcinoma” |
| --- | --- |
| Topic 2  Quality indicators | MeSH terms - Quality Improvement (MeSH) OR Quality Assurance, Health Care (MeSH) OR Quality Indicators, Health Care (MeSH).  String terms - “quality indicator*” OR “process measure*” OR “outcome measure*” OR “performance indicator*” OR “benchmark*" |

Date range 1/1/14 – 30/12/23

Limited to studies in English

Limited to peer-reviewed journal articles

Exclusion criteria:

- Studies on colorectal cancer screening, early stage disease treated with definitive surgery or chemoradiotherapy or surveillance strategies
- Studies that focus on non-clinical QIs (e.g. measures on cost, resource availability, workload, organization)
- Studies focused on generic Qis not applicable to colorectal cancer
- Studies which were not peer-reviewed (case reports, letters, abstracts or editorials)
